# The Quest for Orthologs orthology benchmark service in 2022

**DOI:** 10.1093/nar/gkac330

**Published:** 2022-05-12

**Authors:** Yannis Nevers, Tamsin E M Jones, Dushyanth Jyothi, Bethan Yates, Meritxell Ferret, Laura Portell-Silva, Laia Codo, Salvatore Cosentino, Marina Marcet-Houben, Anna Vlasova, Laetitia Poidevin, Arnaud Kress, Mark Hickman, Emma Persson, Ivana Piližota, Cristina Guijarro-Clarke, the OpenEBench team the Quest for Orthologs Consortium, Adrian Altenhoff, Elspeth A Bruford, Salvatore Cosentino, Christophe Dessimoz, Ingo Ebersberger, David M Emms, Toni Gabaldón, Natasha Glover, Cristina Guijarro-Clarke, Mark Hickman, Yanhui Hu, Wataru Iwasaki, Tamsin E M Jones, Dushyanth Jyothi, Arnaud Kress, Odile Lecompte, Benjamin Linard, Marina Marcet-Houben, Maria J Martin, Yannis Nevers, Emma Persson, Ivana Piližota, Laetitia Poidevin, David S Roos, Erik Sonhammer, Paul D Thomas, David Thybert, Klaas Vandepoele, Anna Vlasova, Bethan Yates, Salvador Capella-Gutierrez, Laia Codó, Meritxell Ferret, Asier Gonzalez-Uriarte, Javier Garrayo-Ventas, Laura Portell-Silva, Dmitry Repchevsky, Vicky Sundesha, Wataru Iwasaki, Odile Lecompte, Erik Sonnhammer, David S Roos, Toni Gabaldón, David Thybert, Paul D Thomas, Yanhui Hu, David M Emms, Elspeth Bruford, Salvador Capella-Gutierrez, Maria J Martin, Christophe Dessimoz, Adrian Altenhoff

**Affiliations:** Department of Computational Biology, University of Lausanne, Lausanne, Switzerland; Swiss Institute for Bioinformatics, University of Lausanne, Lausanne, Switzerland; HUGO Gene Nomenclature Committee (HGNC), European Molecular Biology Laboratory, European Bioinformatics Institute, Wellcome Genome Campus, Hinxton, UK; Protein Function development, European Molecular Biology Laboratory, European Bioinformatics Institute, Wellcome Genome Campus, Hinxton, UK; HUGO Gene Nomenclature Committee (HGNC), European Molecular Biology Laboratory, European Bioinformatics Institute, Wellcome Genome Campus, Hinxton, UK; Barcelona Supercomputing Centre (BSC-CNS). Plaça Eusebi Güell, 1-3 08034 Barcelona, Spain; Barcelona Supercomputing Centre (BSC-CNS). Plaça Eusebi Güell, 1-3 08034 Barcelona, Spain; Barcelona Supercomputing Centre (BSC-CNS). Plaça Eusebi Güell, 1-3 08034 Barcelona, Spain; Department of Biological Sciences, Graduate School of Science, the University of Tokyo, Tokyo, Japan; Barcelona Supercomputing Centre (BSC-CNS). Plaça Eusebi Güell, 1-3 08034 Barcelona, Spain; Institute for Research in Biomedicine (IRB Barcelona), The Barcelona Institute of Science and Technology, Baldiri Reixac, 10, 08028 Barcelona, Spain; Barcelona Supercomputing Centre (BSC-CNS). Plaça Eusebi Güell, 1-3 08034 Barcelona, Spain; Institute for Research in Biomedicine (IRB Barcelona), The Barcelona Institute of Science and Technology, Baldiri Reixac, 10, 08028 Barcelona, Spain; Department of Computer Science, ICube, UMR 7357, Centre de Recherche en Biomédecine de Strasbourg, University of Strasbourg, CNRS, Strasbourg, France; BiGEst-ICube Platform, ICube, UMR 7357, Centre de Recherche en Biomédecine de Strasbourg, University of Strasbourg, CNRS, Strasbourg, France; Department of Computer Science, ICube, UMR 7357, Centre de Recherche en Biomédecine de Strasbourg, University of Strasbourg, CNRS, Strasbourg, France; BiGEst-ICube Platform, ICube, UMR 7357, Centre de Recherche en Biomédecine de Strasbourg, University of Strasbourg, CNRS, Strasbourg, France; Department of Biology, University of Pennsylvania, Philadelphia, PA 19104, USA; Science for Life Laboratory, Department of Biochemistry and Biophysics, Stockholm University, Solna, Sweden; European Molecular Biology Laboratory, European Bioinformatics Institute, Wellcome Genome Campus, Hinxton, UK; European Molecular Biology Laboratory, European Bioinformatics Institute, Wellcome Genome Campus, Hinxton, UK; Department of Biological Sciences, Graduate School of Science, the University of Tokyo, Tokyo, Japan; Department of Integrated Biosciences, Graduate School of Frontier Sciences, the University of Tokyo, Kashiwa, Japan; Department of Computer Science, ICube, UMR 7357, Centre de Recherche en Biomédecine de Strasbourg, University of Strasbourg, CNRS, Strasbourg, France; Science for Life Laboratory, Department of Biochemistry and Biophysics, Stockholm University, Solna, Sweden; Department of Biology, University of Pennsylvania, Philadelphia, PA 19104, USA; Barcelona Supercomputing Centre (BSC-CNS). Plaça Eusebi Güell, 1-3 08034 Barcelona, Spain; Institute for Research in Biomedicine (IRB Barcelona), The Barcelona Institute of Science and Technology, Baldiri Reixac, 10, 08028 Barcelona, Spain; Catalan Institution for Research and Advanced Studies (ICREA), Barcelona, Spain; Centro de Investigaciones Biomédicas en Red de Enfermedades Infecciosas, Barcelona, Spain; European Molecular Biology Laboratory, European Bioinformatics Institute, Wellcome Genome Campus, Hinxton, UK; Department of Population and Public Health Sciences, University of Southern California, Los Angeles, CA 90032, USA; Department of Genetics, Blavatnik Institute, Harvard Medical School, Harvard University, Boston, MA 02115, USA; Department of Plant Sciences, University of Oxford, Oxford OX1 3RB, UK; HUGO Gene Nomenclature Committee (HGNC), European Molecular Biology Laboratory, European Bioinformatics Institute, Wellcome Genome Campus, Hinxton, UK; Department of Haematology, University of Cambridge School of Clinical Medicine, Cambridge, UK; Barcelona Supercomputing Centre (BSC-CNS). Plaça Eusebi Güell, 1-3 08034 Barcelona, Spain; Protein Function development, European Molecular Biology Laboratory, European Bioinformatics Institute, Wellcome Genome Campus, Hinxton, UK; Department of Computational Biology, University of Lausanne, Lausanne, Switzerland; Swiss Institute for Bioinformatics, University of Lausanne, Lausanne, Switzerland; Department of Computer Science, University College London, London, UK; Centre for Life's Origins and Evolution, Department of Genetics, Evolution and Environment, University College London, London, UK; Swiss Institute for Bioinformatics, University of Lausanne, Lausanne, Switzerland; Computer Science Department, ETH Zurich, Zurich, Switzerland

## Abstract

The Orthology Benchmark Service (https://orthology.benchmarkservice.org) is the gold standard for orthology inference evaluation, supported and maintained by the Quest for Orthologs consortium. It is an essential resource to compare existing and new methods of orthology inference (the bedrock for many comparative genomics and phylogenetic analysis) over a standard dataset and through common procedures. The Quest for Orthologs Consortium is dedicated to maintaining the resource up to date, through regular updates of the Reference Proteomes and increasingly accessible data through the OpenEBench platform. For this update, we have added a new benchmark based on curated orthology assertion from the Vertebrate Gene Nomenclature Committee, and provided an example meta-analysis of the public predictions present on the platform.

## Graphical Abstract

**Graphical Abstract:**
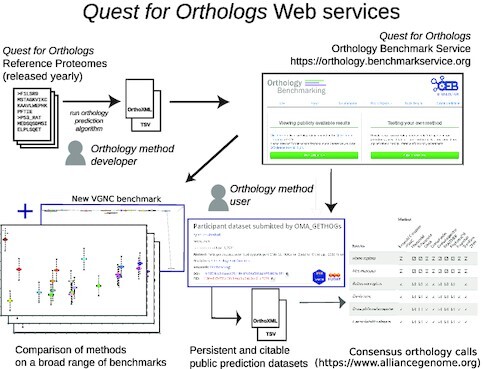
The Quest for Orthologs benchmarking service helps orthology inference methods developers compare their methods on standardized benchmarks, users to choose appropriate methods and provides data for consensus orthology calls.

## INTRODUCTION

Orthology inference—the process of identifying genes originated from the same common ancestor through a speciation event (1)—is a cornerstone of comparative genomics and phylogenetics (2). Inferring orthology is often a difficult process, since the evolutionary histories of gene families may involve multiple duplications, losses, and horizontal transfers of entire genes or individual domains, and because it aims to recapitulate events that took place millions of years ago, under unknown selective pressures, using only the information available from the genomes of modern day species (3). Nevertheless, making such inferences is an important undertaking, central to the process of genome annotation and functional inference, in addition to elucidating evolutionary histories and processes.

The Quest for Orthologs (QfO) consortium is a community effort, bringing together orthology inference software and database developers and their users (4–8). The consortium has been active for >10 years (9), and has continually sought to define common standards shared by diverse stakeholders in the scientific community. One of its most widely recognized efforts is a public benchmarking platform that reports on the performance of several orthology inference methods using various metrics (10).

The benchmarking platform (available at https://orthology.benchmarkservice.org/) has evolved over time to reflect the ever-changing landscape of publicly-available orthology inference methods. Specifically, we aim to make use of incremental improvements in reference genomic datasets and leverage curated datasets that may be used as a proxy for ground truth (10,11). In this paper, we report updates made to the benchmarking platform during the last two years. We describe the continuous updates to the Reference Proteomes underlying the platform as well as to the platform itself with a new benchmark to the existing roster and improved data accessibility. We describe the results of 20 public methods on all the benchmarks, providing an additional key on how to interpret the results and include, for the first time, an in-depth meta-analysis of the predictions.

## NEW QFO REFERENCE PROTEOMES (2020 DATASET)

For standardized and fair benchmarking, and for the results of individual inference methods to be comparable, the benchmarking service needs a consensus dataset of proteomes (hereby understood as the collection of canonical protein sequences from every protein-coding gene in a species). The QfO Reference Proteomes (https://www.ebi.ac.uk/reference_proteomes/) have been jointly designed for this task by the QfO community and the UniProtKB database (12), with a focus on including well-annotated species of medical and scientific interest, and on broadly covering the Tree of Life while staying of manageable size for every orthology inference provider. The dataset is updated annually; the latest version (2020) comprises 78 species (48 Eukaryotes, 23 Bacteria and 7 Archaea) based on the UniProtKB 2020_04 release (apart from the *Xenopus tropicalis* (UP000008143) reference proteome from 2020_06 release data). In aggregate, this represents 1 403 509 protein sequences (984 137 canonical protein sequences and 419 372 isoforms)—a ∼7% increase from the 2018 QfO Reference Proteomes dataset (1 311 679 total sequences). Improvements to the 2020 version of the QfO Reference Proteomes are largely due to updated genome assemblies (Supplementary Table S1), improved genome annotation at the source databases (e.g, Ensembl and RefSeq) and manual curation of entries in UniProt. The QfO Reference Proteomes are available for download in various formats: the protein sequences as FASTA and SeqXML files, CDS sequences for most proteins as FASTA files, and, for an increasing number of species, genomic locus coordinates are available in the XML format.

Reference proteome datasets are generated using a gene-centric approach which identifies all protein isoforms for a gene and selects the canonical protein sequence as representative of the set. The generation of these datasets requires a synchronized update effort of the underlying databases that are the source of protein sequences and gene annotations (including the European Nucleotide Archive, Ensembl, RefSeq, and Model Organism Databases). Generating the 2020 dataset posed a challenge, as the *Xenopus tropicalis* annotation was out of sync across these databases. This was updated and the latest dataset was provided as part of the 2020_06 UniProt release and QfO 2020 release. The new QfO 2021 release has been updated to the new *X. tropicalis* genome assembly (v10).

To help identify the changes in reference proteome sequences, we provide a new STATS file, including a summary of changes to the number of records in the canonical FASTA, additional FASTA and gene symbol to UniProt accession (gene2acc) mapping files, along with report of changes to the source genome assembly for a proteome. This helps to identify whether there are drastic changes in numbers for a given species, and also to track changes over a long period of time. For example, in the case of the *Xenopus tropicalis* records (Supplementary Table S2), one can easily notice there is a major change between releases (−58% of entries) and that the changes are due to an assembly/annotation update from the same import source, i.e. Ensembl.

## RESULTS FOR PUBLIC METHODS

The QFO Benchmark service includes 12 different benchmark tests that measure methods’ performance over several proxies of correct orthology inference. The benchmarks can be broadly divided into three categories: species tree discordance test, agreement with reference orthology (gene phylogenies or curated orthologous pairs) and function-based benchmarks. Each benchmark provides a measure for specificity and recall for the predictions provided by the different software. A top performing method lies on the Pareto frontier, which means that they are not outperformed by other methods on both fronts. Typically, all publically released methods will lie on or close to the Pareto frontier: they mostly differ by their tradeoff between sensitivity and specificity. This is in part because making the results public is an opt-in process and methods that would perform poorly are kept private. Expectedly, the results for previously available methods recapitulate the key findings of previous years: BBH (13) and RSD (14), two naive methods for inferring one-to-one orthologs, were generally outperformed by the publicly available orthology inference algorithms, and no methods consistently outperformed the others across the benchmark.

The benchmarking services have settled on a two years rotation to update its public results, in order to allow time for the main orthology resources and software to provide orthology predictions on each new proteome set, which can consume a lot of computing time. The latest public results are available for the 2020 proteome database on the benchmarking service website (https://orthology.benchmarkservice.org/proxy/projects/2020/). It covers 20 orthology prediction sets coming from 11 different providers - one single resource can provide different types of orthologs, or a software can be run with multiple settings. As evidence of commitment to an updated server, most resources included in the 2020 benchmark already provided inference for the 2018 installment (11), and this year's results include two additional methods - OrthoMCL (15) and Domainoid (16).

Nevertheless, there are qualitative differences between the different methods, in particular regarding the type of orthology inference they aim to produce, which impacts their ranking in the benchmark. In Table 1, we provide a summary of the difference between the methods included in the 2020 public benchmark, their main algorithmic category, the type of orthologous relations they produce, and what is their usual performance in the benchmark. A more detailed comparison of orthology inference methods and resources is available in (2,3). The relationship between type of output and performance is clear for the few methods and algorithms which only aim to provide one-to-one orthology inference (OMA Groups (17), PANTHER LDO (18), BBH (13) and RSD (14)): they constantly rank as the method with the best accuracy at the expense of recall.

**Table 1.** Public methods in the QfO Benchmark 2020. Every method is based on different methodological premises (Described in (3) and detailed on the Benchmarking Service: https://orthology.benchmarkservice.org/proxy/projects/2020/) and provides one or multiple kinds of orthologous prediction. **One-to-one orthologous pairs** (orthologous relationships between single genes), **co-orthologous pairs** (Pairs of orthologs between species, may involve one or multiple genes in the same species depending on duplication), **Orthologous clusters** (a group of orthologs or paralogs defined at one taxonomic level and computed by a graph clustering method), **Hierarchical orthologous groups** (nested groups of orthologs at different taxonomic levels) and **Gene Trees**. The performance column is an indicator based on the usual performance across all benchmarks. Line coloring is tied to the ‘Performance’ column

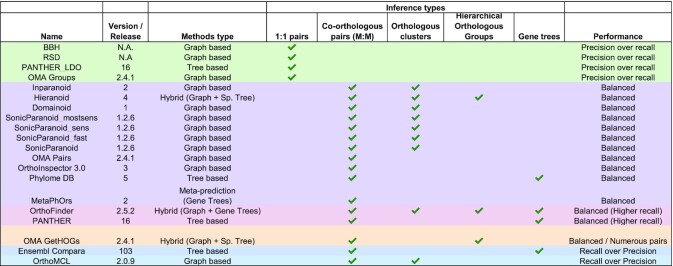

As was noted in previous years, there is not a clear binary distinction in performance between graph-based and tree-based methods. Still, most graph-based methods that primarily aim to provide pairwise orthology inference (including one-to-many, many-to-many and paralogous relationships) tend to rank similarly and cluster together, generally close to the Pareto frontier at the balance between precision and recall. This is also the case of MetaPhOrs (19), a meta-predictor based on multiple gene tree databases that also provide mainly orthologous and paralogous relationships, and PhylomeDB (20), a tree-based method using the species overlap (21) algorithm to reconstruct orthologous relations. PANTHER (22) and OrthoFinder (23), both methods that ultimately aim to provide gene trees, also rank within this ‘balanced’ cluster, though generally with a slight bias toward higher recall (and lower precision). OrthoMCL (15), a graph-based method which computes homologous clusters, often performs among those with the highest recall, though with lower accuracy than other methods. In that, it resembles Ensembl Compara (24), a tree-based method.

It must be noted that while the above paragraphs describe visually assessed trends in ranking over all benchmarks, the result differs from benchmark to benchmark and the reader is invited to refer to them for more details (An in-depth description of individual benchmark methods is available in (10) and (11)). A global overview of the results of all methods across all benchmarks can be found as a summary table in the ‘Public results’ section of the website. Methods may yield different trends depending on the measure used in the benchmark: OMA GETHOGs (25) is a good example of this, ranking close to Ensembl Compara with high recall and lower accuracy in the functional benchmark (due to the high number of orthologous pairs inferred), but closer to OMA Pairs in the reference gene tree benchmark, in the ‘balanced’ cluster.

## NEW VGNC BENCHMARK

For the 2020 issue of the benchmarking service, we supplemented the 11 benchmarks from previous years with a new one based on curated gene symbols from the Vertebrate Gene Nomenclature Committee (VGNC) (26) to evaluate pairwise orthology inference.

The VGNC defines a standardized nomenclature for genes in vertebrate species, and an orthologous relationship between genes is a required condition to propagate a gene symbol across species. For this new benchmark, the VGNC provided us with a list of human-curated sets of orthologous relations between genes, including gene families curated by experts with complicated evolutionary histories such as Olfactory Receptors (27) and Cytochrome P450s. In order to avoid circularity, these relations are only ones defined through human curation and not on the basis of automated orthology inference software. In total, the dataset covers 18,282 sets of orthologous genes from eight mammalian species (*Homo sapiens*, *Pan troglodytes, Macaca mulatta, Bos taurus, Canis lupus familiaris, Felis catus, Equus caballus* and *Sus scrofa*), the first four of which are included in the QFO dataset and can be used for benchmarking. An orthologous set includes at most one gene from each species, where each gene is orthologous to each other. We extracted every pair of genes within a group and considered them as true orthologous pairs. The benchmark can be interpreted as an assessment of how good methods are in correctly calling recently diverged orthologous pairs, even in difficult gene families.

We use the True Positive Rate (TPR), the proportion of pairs successfully retrieved by a method, as a measure of recall, and for precision we use the Positive Predictive Value (PPV). Here, we consider as a False Positive any pair inferred between genes from different orthologous sets, when these orthologous sets already have a gene for both species in the pair.

**Figure 1.**
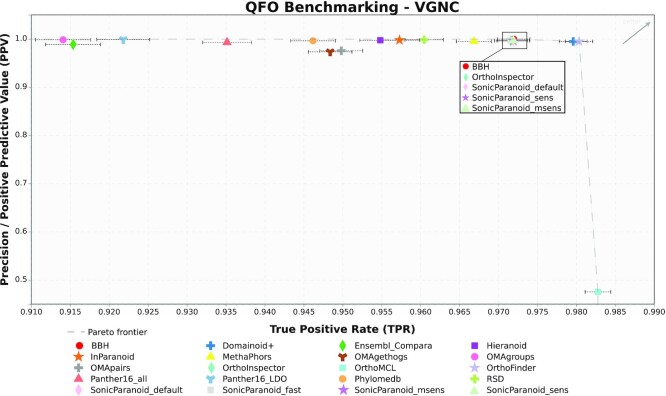
VGNC symbol Benchmark. Results of each method with True Positive Rate (x-axis) as a recall measure and Positive Predictive Value (y-axis) as a precision measure. Note: The axes have been truncated to maximize the spread of the methods on the figure. As a result, the y-axis shows a significantly larger variation in values than the x-axis

The result is shown in Figure 1. As expected, considering that the dataset concerns orthology relations between mammalian species, most methods perform well on the precision front, with a PPV higher than 0.95 for all but one method. They also perform well on the recall side, where all methods achieve at least 0.9 of TPR. OMA Groups and PANTHER LDO achieve the best precision (PPV: 0.996) with slightly lower recall (TPR: 0.914 and 0.922 respectively) than other methods while OrthoMCL has the best recall but at the cost of a PPV being more than half of the other methods (PPV:0.443, TPR:0.983). Most other methods are on or close to the Pareto Frontier between the two extremes, keeping a PPV close to 1 and varying mostly on recall. Domainoid and OrthoFinder are the two methods that appear to get the best trade-off in this regard, keeping their PPV close to 1 (respectively 0.990 and 0.988) while reaching a TPR of 0.980.

## ADDING VALUE TO QfO SUBMITTED DATASETS VIA OpenEBench AND EUDAT B2SHARE

Stable long-term data sharing is fundamental for guaranteeing results reproducibility and enabling data reusability in downstream analyses. Indeed, for the 2020 installment, OpenEBench (28) has upgraded its underlying data infrastructure. Specifically, OpenEBench now makes use of B2SHARE (https://b2share.eudat.eu/) for long-term availability and storage of publicly available QfO datasets. B2SHARE is the repository service for sharing research data of the EUDAT Data Collaborative Infrastructure (https://eudat.eu/), one of the largest e-infrastructures in Europe offering permanent storage capacity and integrated management services for research communities. Access to EUDAT records is possible through the links on the Public Projects page of the Benchmarking service, by clicking on the Uploaded data link. There one can access the metadata and download the predicted orthologous pairs. One of the key reasons for adopting the EUDAT B2SHARE technical standards, data models and policies is that it helps OpenEBench to further enforce its FAIR data principles (29) *by design* compliant strategy. Indeed, when using B2SHARE, QfO datasets become on one side *Findable*, through the assignment of permanent identifiers, as Digital Object Identifiers (DOI), and on the other *Accessible*, through the EUDAT infrastructure, adding value and visibility to QfO participant submission. Importantly, ownership is always preserved, as OpenEBench acts on behalf of the participant.

Beyond QfO public orthology prediction, OpenEBench includes a variety of dataset types as part of the evaluation benchmarking workflows. Those datasets cover the reference data used in benchmarking events as gold standards, the already mentioned submitted predictions and, also, the actual results scoring and comparing participants, provenance reports and metrics comparison plots. In this way, a compact and human-readable set of data is ready to be referred to in scientific publications, promoting transparency, reproducibility and data reuse. Furthermore, B2SHARE records’ registries provide rich metadata fields for easing data discovery, so thus submitted collections could be annotated with cross-links to OpenEBench for further insights. To reflect this new functionality and other improvements, the OpenEBench documentation (https://openebench.readthedocs.io/) has been extensively rewritten, facilitating the platform usage according to the different user profiles.

## META-ANALYSES OF PUBLIC INFERENCE METHODS

As the benchmarking service requires every public method to make their inferences on a standardized dataset, the publicly available datasets can be easily used as a source for meta-prediction of orthologous relationships across multiple orthology inference methods. This has multiple advantages: distant orthology relations may not be unveiled by all inference methods, thus diversifying the source of prediction makes it more likely one could discover such pairs. On the other hand, if a user is interested in relying only on the most robust orthology inferences, the number of methods that infer any orthologous pair can be used as a confidence score. This aggregation of orthology inference methods is already made by several meta-prediction resources: HGNC Comparison of Orthology Predictions (30,31), DIOPT (32), OrthoList (33) or WORMHOLE (34). The public predictions directly downloadable from the QfO benchmarking service are first processed by the DIOPT pipeline, integrated with manually curated orthology resources such as HGNC, ZFIN and SGD, then used by the Alliance of Genome Resources as part of their orthologous pairs meta-predictor. We encourage other meta-prediction method developers to download pairs from the platform.

**Figure 2.**
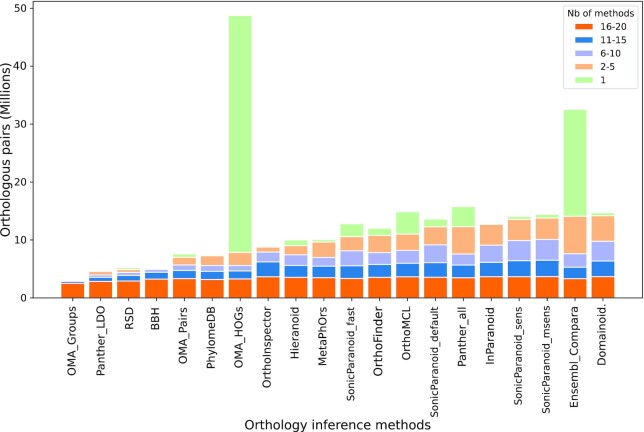
Orthologous pairs inferred by individual methods. Number of pairs inferred by public methods included in the benchmarking platform. Subsections of the bars represent the number of methods that share the same pairs. Methods are ranked by the number of pairs they share with other methods (non-green part of the stacked bars).

The comparisons of pairs predicted by the different methods can also be used as a way to better understand the differences and similarities between orthology inference methods. In Figure 2, we break down the prediction of every individual inference method by the number of other methods that recapitulate the same orthologous pairs. This breakdown allows to shed a different light onto the results obtained by the different benchmarks. The methods that aim to only infer one-to-one orthologous pairs yield the smaller number of predictions, a high proportion of which are also inferred by most other methods: this is in accordance with their general performance of high specificity and lower recall seen in the benchmark. There is a sizable set of high confidence pairwise orthologous pairs, around 3 million, that are shared between more than 15 methods. However, it may be surprising that for most public methods that aim to provide co-orthologous pairs, around half of the predictions are shared by less than ten methods overall, suggesting that there is enough difference between all the public predictions that a wider consensus is hard to reach.

The two most sensitive versions of SonicParanoid, Domainoid, and Ensembl Compara are the methods that output the highest number of pairs predicted by at least one of the other methods. However, for Domainoid and SonicParanoid, this is affected by the fact that there is at least one other public prediction in the dataset based on similar methodological premises. Accordingly, when we repeat this analysis by considering only one of the redundant sets, the number of ‘shared pairs’ from these two methods diminishes, getting them closer to PANTHER, OrthoFinder and OrthoMCL (Supplementary Figure S1). Few methods provide a high number of pairs that are specific to their own methods, but interestingly and perhaps expectedly, the methods that provide the highest number of ‘method-specific’ predictions (Ensembl Compara and OMA GETHOGs) correspond to the methods that generally exhibit higher recall, at the cost of precision. Conversely, the methods that tend to strike a balance between both recall and precision mostly recover pairs that are predicted by at least one other method. Interestingly, the especially high volume of pairs predicted by Ensembl and OMA GETHOGs are specific to their own method, meaning there is likely low overlap between these two methods beside a similarity in terms of number of predicted pairs. The reasons behind the comparatively greater number of predictions provided by these two methods are unclear. It likely relates to the attempt of the methods to reconstruct deep many-to-many relations, which results in a high number of pairs in ancient and highly duplicated gene families.

**Figure 3.**
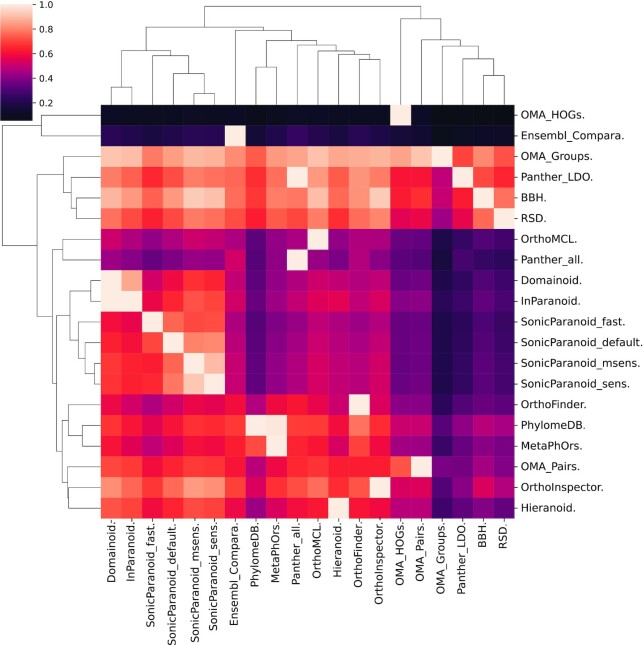
Pairwise comparisons between predictions of public methods. The heatmap shows the proportion of the pairs inferred by methods on the right side that are recapitulated by methods on the bottom. The heatmap is hierarchically clustered on rows and columns by similarity with the corresponding trees shown.

To better grasp the similarity and dissimilarity between methods, we looked at the fraction of the predictions of each method that was shared by any other method in a pairwise comparison (Figure 3). These results reiterate that the predictions provided by the one-to-one orthologous inferences have high overlaps with other methods, while a comparatively low proportion of inferences provided by OMA GETHOGs and Ensembl Compara are retrieved by other methods. In addition, this comparison unveils expected similarities between some of the public methods included in the benchmarks: for example, all predictions made by PANTHER LDO are made by PANTHER, and the same is true for PhylomeDB and MetaPhOrs, as well as for Inparanoid and Domainoid, meaning that in these cases the first set of predictions is a subset of the second. Similarly, the inference sets from Inparanoid, Domainoid, and SonicParanoid run with different parameters have high overlap (but not Hieranoid), as expected from methods based on very similar approaches.

This surface analysis of the inference prediction methods is only an example of what the benchmarking service platform, and its freely available data from public methods, makes possible in terms of additional assessments of the inference set provided by every method on a common dataset. This kind of overlap comparison gives additional context on how to better interpret inference from meta-prediction and on what methods use or include as part of a custom meta-predictor.

## PERSPECTIVES

After >5 years of continued service, the Benchmarking platform continues to be widely used and plays its role in evaluating the performance of the existing orthology software and databases. This is made possible by a commitment from the community to provide regularly updated data (orthology prediction with different methods) and by the incremental improvement to the benchmark over the years. In this year's update, the improvement took the form of new benchmarks, an increasing traceability in the update of the reference proteomes, and easier accessibility of public data, which opens the path to more efficient data sharing outside of the community.

The current Quest for Orthologs benchmark is designed to accommodate a variety of orthologous benchmarks, yielding a diversity of orthologous inferences. Thus, the legacy benchmark data type has been the orthologous pairs: a type of prediction that can be made by all methods. Yet, most comparative genomics studies done today aim to consider orthologous relations across a whole set of species, at the gene families level, an endeavor that is only made possible by hierarchical orthologous groups and gene tree predictions. In the short term, we aim to start including a group-based benchmark to the benchmarking service and, as a first example, we implemented a version of the OrthoBench benchmark (35,36) to the benchmarking service. However, integrating group-based benchmarks into the benchmarking service necessitates solving additional challenges. First, we will work on an updated platform to be able to handle independently the universal pair-based benchmark and the narrower orthologous groups benchmark, which can be applied only to a subset of methods. Second, community efforts will be directed into facilitating the adoption of the OrthoXML (37) format for orthogroups or gene tree inference provider as a common format to describe the nature of these relations.

Properly benchmarking orthology inference methods is challenging due to the absence of an universal ground truth to unambiguously validate or invalidate the predicted orthologous relations. The benchmarking service has been designed to make up for this by assessing methods performance over several independent benchmarks making use of different proxies. The newly implemented VGNC benchmark has been added under the same logic: making use of high-confidence orthologous pairs curated by experts. Because of its nature, the benchmark is mainly informative for assessing the capacity of the methods to recapitulate accurate orthologous pairs between closely related species, under different degrees of complexity of evolutionary history. Though most methods perform well on the benchmark with high recall and precision, their relative ranking is indicative of how well they are able to solve the most difficult cases in comparison to others. The measures used to evaluate performance, especially accuracy, are non-trivial to implement because of the absence of ‘negative set’ in the dataset. Our choice here was to use a conservative definition of what is called a false prediction rather than risk wrongly penalizing a method for a real prediction. Thus, users are invited to refer to the relative ranking error interval rather than absolute value only when using the benchmark. The goal of the Benchmarking Service will be to continue, whenever possible, to integrate new benchmarks using curated data when it has the potential to add valuable information about the performance of the public methods on different conditions. We invite the broader community to reach out to the consortium if they believe other dataset could be used for this endeavor in the future

Despite there being several methods trying to provide orthology inference, the meta-analysis provided here shows that the agreement level between their predictions is not necessarily high. Partly, it comes down to the stated goal of the methods: at one end of the spectrum, some aim to provide fewer, high confidence orthology calls, while others aim to identify more orthologous relations with some impact on precision. Users should weigh these differences when selecting orthology inference methods, and select the one most adapted to their purpose. The different benchmarks provided by the orthology benchmark platform should give them additional information in which context it is optimal to use one method over another. For example, users interested in function propagation may want to choose a method performing best on function related benchmarks while others interested in constructing gene trees for particular gene families should look at the gene trees related benchmarks. In some cases, using a combination of methodologically dissimilar inference methods could be the best course of action, allowing to explore a higher space of orthologous relations and to use the number of convergent orthology calls from different methods as confidence scores.

## DATA AVAILABILITY

Public benchmark results for the latest release are available on the orthology benchmarking service website (https://orthology.benchmarkservice.org) under the “Public results” section, “QfO Benchmark release 2020” subsection. Public orthologous pair predictions can be downloaded from the EUDAT platform through links under the “Public projects”.

## Supplementary Material

gkac330_Supplemental_File

## APPENDIX

**THE QUEST FOR ORTHOLOGS CONSORTIUM MEMBERS:**

Adrian Altenhoff^2,5^, Elspeth A. Bruford^15,16^, Salvatore Cosentino^6^, Christophe Dessimoz^1,2,3,4^, Ingo Ebersberger^23,24,25^, David M. Emms^19^, Toni Gabaldón^9,10,11,12^, Natasha Glover^1,2^, Cristina Guijarro-Clarke^18^, Mark Hickman^13^, Yanhui Hu^21^, Wataru Iwasaki^6,7^, Tamsin E.M. Jones^15^, Dushyanth Jyothi^17^, Arnaud Kress^8,22^, Odile Lecompte^8^, Benjamin Linard^26^, Marina Marcet-Houben^9,10^, Maria J. Martin^17^, Yannis Nevers^1,2^, Emma Persson^14^, Ivana Piližota^18^, Laetitia Poidevin^8,22^, David S. Roos^13^, Erik Sonhammer^14^, Paul D. Thomas^20^, David Thybert^18^, Klaas Vandepoele^27,28,29^, Anna Vlasova^9,10^, Bethan Yates^15^

**THE OPEN EBENCH TEAM:**

Salvador Capella-Gutierrez^9^, Laia Codó^9^, Meritxell Ferret^9^, Asier Gonzalez-Uriarte^9^, Javier Garrayo-Ventas^9^, Laura Portell-Silva^9^, Dmitry Repchevsky^9^, Vicky Sundesha^9^

1. Department of Computational Biology, University of Lausanne, Lausanne, Switzerland
2. Swiss Institute for Bioinformatics, University of Lausanne, Lausanne Switzerland
3. Department of Computer Science, University College London, London, United Kingdom
4. Centre for Life's Origins and Evolution, Department of Genetics, Evolution and Environment, University College London, London, United Kingdom
5. Computer Science Department, ETH Zurich, Zurich, Switzerland
6. Department of Biological Sciences, Graduate School of Science, the University of Tokyo, Tokyo, Japan
7. Department of Integrated Biosciences, Graduate School of Frontier Sciences, the University of Tokyo, Kashiwa, Japan
8. Department of Computer Science, ICube, UMR 7357, Centre de Recherche en Biomédecine de Strasbourg, University of Strasbourg, CNRS, Strasbourg, France
9. Barcelona Supercomputing Centre (BSC-CNS). Plaça Eusebi Güell, 1–3 08034 Barcelona, Spain.
10. Institute for Research in Biomedicine (IRB Barcelona), The Barcelona Institute of Science and Technology, Baldiri Reixac, 10, 08028 Barcelona, Spain.
11. Catalan Institution for Research and Advanced Studies (ICREA), Barcelona, Spain.
12. Centro de Investigaciones Biomédicas en Red de Enfermedades Infecciosas, Barcelona, Spain.
13. Department of Biology, University of Pennsylvania, Philadelphia, PA 19104, USA.
14. Science for Life Laboratory, Department of Biochemistry and Biophysics, Stockholm University, Solna, Sweden
15. HUGO Gene Nomenclature Committee (HGNC), European Molecular Biology Laboratory, European Bioinformatics Institute, Wellcome Genome Campus, Hinxton, United Kingdom
16. Department of Haematology, University of Cambridge School of Clinical Medicine, Cambridge, United Kingdom
17. Protein Function development, European Molecular Biology Laboratory, European Bioinformatics Institute, Wellcome Genome Campus, Hinxton, United Kingdom
18. European Molecular Biology Laboratory, European Bioinformatics Institute, Wellcome Genome Campus, Hinxton, United Kingdom
19. Department of Plant Sciences, University of Oxford, Oxford, OX1 3RB, UK
20. Department of Population and Public Health Sciences, University of Southern California, Los Angeles, CA 90032, USA
21. Department of Genetics, Blavatnik Institute, Harvard Medical School, Harvard University, Boston, MA 02115, USA.
22. BiGEst-ICube Platform, ICube, UMR 7357, Centre de Recherche en Biomédecine de Strasbourg, University of Strasbourg, CNRS, Strasbourg, France
23. Institute of Cell Biology and Neuroscience, Goethe University Frankfurt, Frankfurt, Germany.
24. Senckenberg Biodiversity and Climate Research Centre (S-BIKF), Frankfurt, Germany.
25. LOEWE Center for Translational Biodiversity Genomics (TBG), Frankfurt, Germany.
26. SPYGEN, Le Bourget-du-Lac, France.
27. Department of Plant Biotechnology and Bioinformatics, Ghent University, Ghent, Belgium.
28. VIB Center for Plant Systems Biology, Ghent, Belgium.
29. Bioinformatics Institute Ghent, Ghent University, Ghent, Belgium.
