# Supplementary material for: The Quest for Orthologs orthology benchmark service in 2022
Source: Nucleic Acids Res. 2022 May 12;50(W1):W623–32. doi: 10.1093/nar/gkac330 (PMC9252809; doi:10.1093/nar/gkac330)
Supplement: gkac330_Supplemental_File [file gkac330_supplemental_file.pdf]

Supplementary Material

Proteome UPID	Organism	2018_04	2020_04
UP000000437	Danio rerio (Zebrafish)	GCA_000002035.3	GCA_000002035.4
UP000000539	Gallus gallus (Chicken)	GCA_000002315.3	GCA_000002315.5
UP000000589	Mus musculus (Mouse)	GCA_000001635.7	GCA_000001635.8
UP000001450	Plasmodium falciparum (isolate 3D7)	GCA_000002765.2	GCA_000002765.3
UP000001548	Giardia intestinalis (strain ATCC 50803 / WB clone C6)	GCA_000002435.1	GCA_000002435.2
UP000005640	Homo sapiens (Human)	GCA_000001405.25	GCA_000001405.27
UP000006548	Arabidopsis thaliana (Mouse-ear cress)	GCA_000001735.1	GCA_000001735.2
UP000009136	Bos taurus (Bovine)	GCA_000003055.3	GCA_002263795.2

Supplementary Table 1. Assembly updates in QFO Reference Proteomes. All proteomes for which the reference assembly changed from the last benchmark release and corresponding version numbers.

Proteome_ID	UP000008143
Tax_ID	8364
#(1) Number of entries in main fasta (canonical) [last qfo release number of entries, difference between current and last number of entries, percent change between current and last release]	23799 (prev:37604, diff:-13805, %change:-58)
#(2) Number of entries in additional fasta (isoforms) [last qfo release number of entries, difference between current and last number of entries, percent change between current and last release]	23799 (prev:37604, diff:-13805, %change:-58)
#(3) Number of entries in gene2acc	6603 (prev:57261, diff:-10658,

mapping file [last qfo release number of entries, difference between current and last number of entries, percent change between current and last release]	%change:-22)
Assembly(Previous_Assembly)	GCA_000004195.4 (GCA_000004195.1)
Source(Previous_Source)	Ensembl (Ensembl)
Species_Name	Xenopus tropicalis (Western clawed frog) (Silurana tropicalis)

Supplementary Table 2. **STATS file for *Xenopus tropicalis* (2020 Release).** The STAT file reports change in number of entries in proteomes between releases, as well as differences in assembly and provider.

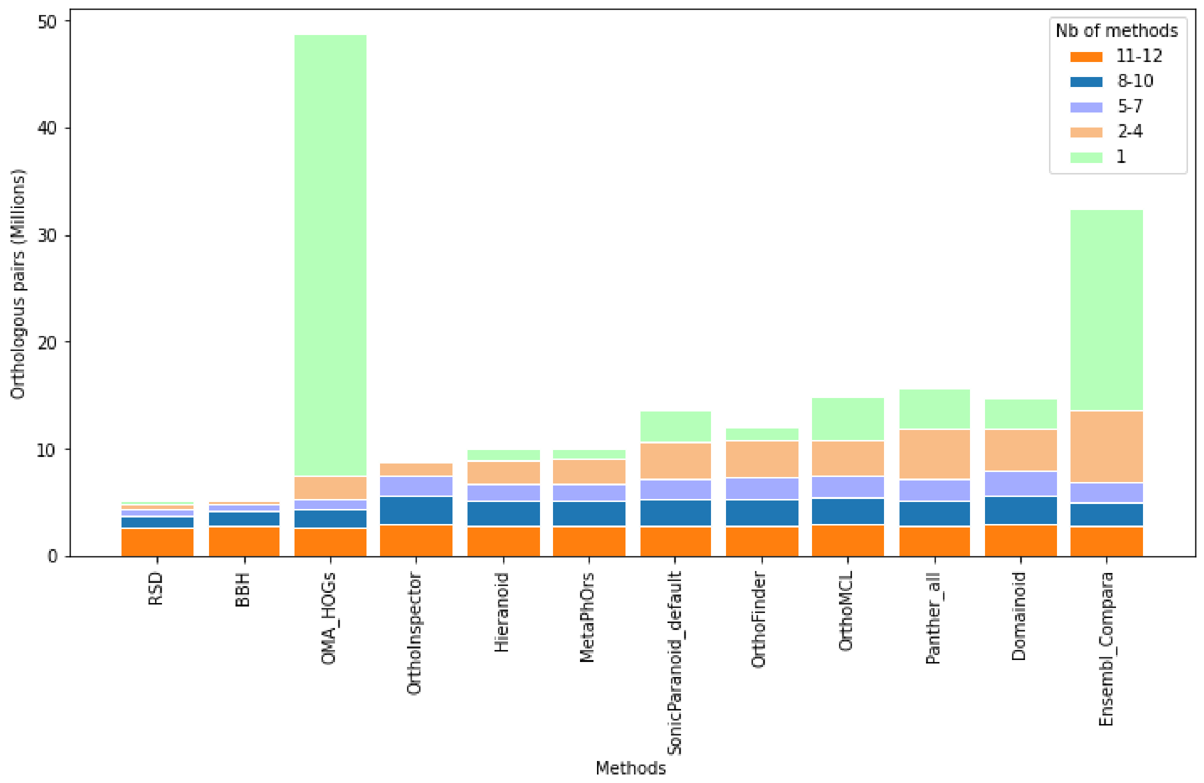

Supplementary Figure 1. Orthologous pairs inferred by individual non-redundant methods. Number of pairs inferred by public methods included in the benchmarking platform. Subsections of the bars represent the number of methods that share the same pairs. Methods are ranked by the number of pairs they share with other methods (non-green part of the stacked bars). Only one method was selected for methods based on the same approach, as follow: OMA_HOGs is representative for OMA predictions (OMA_Pairs, OMA_Groups, OMA_HOGs), PANTHER_all is representative of PANTHER predictions (PANTHER_all, PANTHER_LDO), Domainoid is chosen as representative of Inparanoid-based predictions (Inparanoid, Domainoid) and SonicParanoid_default is chosen as representative of Sonicparanoid predictions (SonicParanoid_default, SonicParanoid_fast, SonicParanoid_sens, SonicParanoid_msens).
